# The Association Between Serum Trimethylamine *N*-Oxide and Arterial Stiffness in Chronic Peritoneal Dialysis Patients: A Cross-Sectional Study

**DOI:** 10.3390/toxins16120523

**Published:** 2024-12-03

**Authors:** Po-Yu Huang, Yu-Li Lin, Yi-Hsin Chen, Szu-Chun Hung, Hung-Hsiang Liou, Jen-Pi Tsai, Bang-Gee Hsu

**Affiliations:** 1 Institute of Medical Sciences, Tzu Chi University, Hualien 97004, Taiwan; poyuhs13628@gmail.com (P.-Y.H.); nomo8931126@gmail.com (Y.-L.L.); 2 Division of Nephrology, Department of Internal Medicine, Dalin Tzu Chi Hospital, Buddhist Tzu Chi Medical Foundation, Chiayi 62247, Taiwan; 3 Division of Nephrology, Hualien Tzu Chi Hospital, Buddhist Tzu Chi Medical Foundation, Hualien 97004, Taiwan; 4 School of Medicine, Tzu Chi University, Hualien 97004, Taiwan; nephp06@gmail.com (Y.-H.C.); szuchun.hung@gmail.com (S.-C.H.); 5 Division of Nephrology, Department of Internal Medicine, Taichung Tzu Chi Hospital, Buddhist Tzu Chi Medical Foundation, Taichung 40201, Taiwan; 6 Division of Nephrology, Department of Internal Medicine, Taipei Tzu Chi Hospital, Buddhist Tzu Chi Medical Foundation, Taipei 23142, Taiwan; 7 Division of Nephrology, Department of Internal Medicine, Hsin-Jen Hospital, New Taipei City 24243, Taiwan; hh258527@ms23.hinet.net

**Keywords:** carotid–femoral pulse wave velocity, trimethylamine *N*-oxide, peritoneal dialysis, arterial stiffness

## Abstract

Trimethylamine *N*-oxide (TMAO), a gut microbiome-derived metabolite, participates in the atherogenesis and vascular stiffening that is closely linked with cardiovascular (CV) complications and related deaths in individuals with kidney failure undergoing peritoneal dialysis (PD) therapy. In these patients, arterial stiffness (AS) is also an indicator of adverse CV outcomes. This study assessed the correlation between serum TMAO concentration quantified with high-performance liquid chromatography and mass spectrometry and central AS measured by carotid–femoral pulse wave velocity (cfPWV) in patients with chronic PD. Of the 160 participants included, 23.8% had a cfPWV of ≥10 m/s, which fulfilled the AS criteria. Multivariable logistic regression analysis revealed that TMAO, age, and waist circumference were positively associated with AS. Multivariable stepwise linear regression showed that underlying diabetes, advanced age, waist circumference, systolic blood pressure, and logarithmic-transformed TMAO were independently correlated with cfPWV. The area under the receiver operating characteristic curve for TMAO in differentiating AS from non-AS was 0.737. In conclusion, serum TMAO level was significantly independently correlated with central AS among participants undergoing PD for end-stage kidney failure.

## 1. Introduction

A substantial proportion of individuals with kidney failure requiring dialysis therapy have cardiovascular (CV) disorders [1]. Moreover, the risk of mortality attributed to CV conditions is significantly greater in patients having end-stage kidney disease (ESKD) than in those without kidney failure [2,3]. According to the 2021 Annual Report of the Taiwan Renal Data System, the five-year cumulative survival probability of patients undergoing maintenance peritoneal dialysis (PD) was 65.9% [4]. Loss of residual kidney function, extracellular fluid volume excess, lipid disorders, disturbed calcium and phosphorus balance, decreased insulin sensitivity, chronic inflammation, and genetic modifications predispose patients with PD to adverse CV consequences [5,6].

Arterial stiffness (AS) is the result of anatomical and functional derangements within the arterial wall [7], and its degree is influenced by genetic factors, physiologic aging, and various disease states, such as high blood pressure, hyperinsulinemia, kidney dysfunction, and chronic inflammation [8,9]. Central AS is primarily estimated by carotid–femoral pulse wave velocity (cfPWV) [10]. A cohort study performed in China has proven the value of cfPWV in the estimation of the ten-year risk of atherosclerotic CV disease [11]. A systematic review and meta-analysis also concluded that cfPWV was associated with a greater chance of CV-related mortality and overall mortality [12]. Relatively high pulse wave velocities in patients with chronic kidney disease (CKD) can predict a greater risk of progression to ESKD and death from any etiology [13]. In patients with ESKD, arteriosclerosis worsens due to hypervolemia, osteoblastic transformation of smooth muscle cells, oxidative stress, and upregulation of angiopoietin 2 [14].

Trimethylamine *N*-oxide (TMAO), a well-recognized uremic molecule originating from the gastrointestinal tract, is implicated in atherosclerotic disease pathophysiology and can predict adverse CV outcomes [15]. Trimethylamine is synthesized by intestinal microorganisms through the metabolism of choline, L-carnitine, and betaine and is then absorbed and further transformed into TMAO by hepatic enzymes [16]. TMAO is primarily excreted through the kidneys, and there is an inverse relationship between serum TMAO level and glomerular filtration rate; therefore, TMAO can be regarded as an endogenous marker for estimating kidney function [17]. Furthermore, previous meta-analyses have demonstrated that relatively high serum TMAO concentrations were correlated with an increased likelihood of major adverse CV events and worse overall prognosis [18,19]. Particularly among individuals with renal insufficiency with or without maintenance dialysis treatment, a systematic review and meta-analysis revealed that serum TMAO was a novel predictor of long-term prognosis in a concentration-dependent manner [20]. In addition, relatively high TMAO values were associated with increased odds of death attributed to CV conditions among patients undergoing PD [21].

A small cross-sectional investigation concluded that in patients with preexisting atherosclerotic CV diseases or at risk of developing CV disorders, serum TMAO levels were significantly associated with cfPWV, but the association was attenuated after adjustment for age, blood pressure, and glycemic status [22]. In our previous publication, TMAO was reported to be independently associated with peripheral AS, which was determined by increased brachial-ankle pulse wave velocities in patients with nondialysis renal failure [23]. In addition, in patients with kidney failure on long-term hemodialysis, TMAO was independently correlated with central AS [24]. However, the role of TMAO in the pathophysiology of vascular stiffening in patients receiving chronic PD remains unclear. Therefore, this clinical study was performed to examine the association between TMAO and AS in this population.

## 2. Results

In total, 160 patients undergoing chronic PD therapy were incorporated in this study, and their baseline characteristics are listed in Table 1. The modalities used were continuous ambulatory PD in 61 patients and automated PD in the remaining patients. Underlying diabetes mellitus (DM) and hypertension were identified in 38.8% and 74.4%, respectively. Overall, the median duration of PD therapy was 46.34 months (interquartile range, 21.03–84.78 months). Of the 160 study participants, 38 were diagnosed as fulfilling the criteria for AS. Compared with patients without the diagnosis of AS, those included in the AS group were significantly older (*p* = 0.005), had significantly larger waist circumference (*p* = 0.005), higher systolic blood pressure (SBP) (*p* = 0.023), higher concentration of fasting plasma glucose (*p* = 0.006), greater proportion of diabetes (*p* = 0.002), and higher serum TMAO concentration (*p* < 0.001); and significantly lower weekly Kt/V value as a classic indicator of overall small solute clearance (*p* = 0.045). The two groups had no significant differences in sex distribution; percentage of patients who were on blood pressure-lowering or antihyperlipidemic prescriptions; body mass index (BMI); prevalence of hypertension; diastolic blood pressure (DBP); peritoneal Kt/V; whole clearance of creatinine per week; weekly peritoneal creatinine clearance; weekly creatinine clearance from residual urine; and blood levels of hemoglobin, total cholesterol, albumin, blood urea nitrogen, creatinine, total calcium ion, inorganic phosphorus, and intact parathyroid hormone (iPTH).

On multivariable logistic regression analysis, the identified parameters that independently correlated with AS were serum TMAO [odds ratio (OR) for every 1 μg/L increase 1.011, 95% confidence interval (CI) 1.005–1.017, *p* < 0.001]; age (OR for every increase in 1 year 1.053, 95% CI 1.013–1.095, *p* = 0.009); and waist circumference (OR for every 1-cm increase 1.047, 95% CI 1.003–1.094, *p* = 0.038) (Table 2).

**Figure 1:**
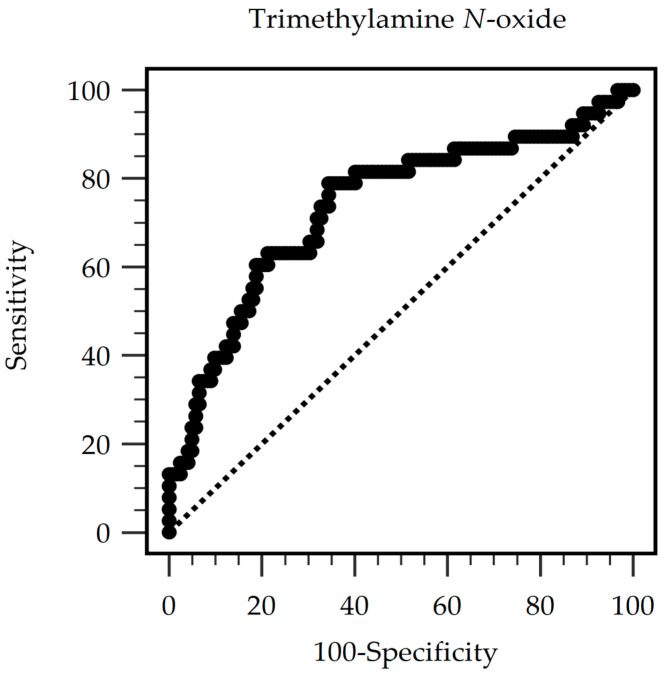
The area under the receiver operating characteristic curve indicates the diagnostic power of trimethylamine *N*-oxide levels for predicting arterial stiffness among peritoneal dialysis patients.

Figure 1 shows the receiver operating characteristic (ROC) curve for the capability of serum TMAO concentration to distinguish between AS and non-AS in large vessels. The area under the curve was 0.737 (95% CI 0.662–0.804, *p* < 0.0001); the best threshold TMAO value was 120.10 µg/L, with 78.95% sensitivity, 65.57% specificity, 41.66% positive predictive value, and 90.91% negative predictive value. We also performed the ROC curve analysis for the waist circumference (area under the curve 0.665, 95% CI 0.586–0.737, *p* = 0.0005) as well as combined TMAO and waist circumference (area under the curve 0.774, 95% CI 0.702–0.837, *p* < 0.0001) (Supplementary Figure S1). There were no significant differences among these areas under the curve.

Table 3 shows this study population’s potential clinical factors correlated with cfPWV. Simple regression analysis showed that the presence of DM (*r* = 0.368, *p* < 0.001); age (*r* = 0.347, *p* < 0.001); BMI (*r* = 0.240, *p* = 0.002); waist circumference (*r* = 0.330, *p* < 0.001); SBP (*r* = 0.295, *p* < 0.001); logarithmically transformed serum glucose (log-glucose, *r* = 0.299, *p* < 0.001); and log-TMAO (*r* = 0.346, *p* < 0.001) were significantly positively related with increased cfPWV values. Multivariable stepwise linear regression analysis of these variables revealed that the independent predictors of high cfPWV were DM (β = 0.163, adjusted R^2^ change = 0.130, *p* = 0.022); advanced age (β = 0.218, adjusted R^2^ change = 0.071, *p* < 0.001); waist circumference (β = 0.256, adjusted R^2^ change = 0.058, *p* < 0.001); SBP (β = 0.208, adjusted R^2^ change = 0.047, *p* = 0.002); and log-TMAO (β = 0.213, adjusted R^2^ change = 0.038, *p* = 0.002). The scatter plots (continuous variables) and boxplots (categorical variables) are shown in Supplementary Figure S2 to further evaluate the relationship between these variables and the cfPWV.

## 3. Discussion

The key result of this research was that serum TMAO concentration, patient age, and waist circumference were independently associated with increased central AS among patients undergoing PD. In addition, a diagnosis of diabetes, age, waist circumference, SBP, and TMAO levels were independently and positively correlated with cfPWV in this population.

In individuals with ESKD, various factors increase the risk of and precipitate AS. Patients with kidney failure have sympathetic overactivity and volume overload, leading to a high prevalence of long-standing hypertension, which results in continued vascular remodeling [25]. Accumulation of uremic molecules in the setting of reduced renal function causes malfunction of the vascular endothelium [26]. Dextrose-rich peritoneal dialysate, acute and chronic infections, and diminished residual kidney function were linked to chronic inflammatory status and vascular damage in patients undergoing PD [27]. CKD mineral and bone disorders manifest as vascular calcification and arteriosclerosis [28].

Gut dysbiosis, which is a condition of disordered intestinal microbiome, contributes to the initiation and propagation of atherosclerosis and has various metabolic consequences [29,30]. TMAO is produced by intestinal bacteria and mediates atherogenesis through aberrant activation of inflammatory pathways, dysregulated cholesterol metabolism, endothelial damage, oxidative stress, and induction of thrombus formation [31,32]. In one study, TMAO treatment of human endothelial cells enhanced the expression of cytokines, chemotactic factors, and adhesion molecules [33]. Another study showed that the signaling transduction pathways associated with nuclear factor kappa B (NF-κB) and p38 mitogen-activated protein kinase were upregulated by TMAO [34]. For thrombogenicity, TMAO activates platelets by inducing calcium flux into the cytosol [35] and regulates tissue factor expression [36]. In addition to its involvement in atherosclerotic plaque formation, TMAO contributes to medial calcification in blood vessels by precipitating calcium and phosphorus and triggering NF-κB- and NLRP3 inflammasome-dependent downstream intracellular signaling [37]. Taken together, these mechanisms influenced by TMAO could substantially make patients vulnerable to adverse CV sequelae.

The association between TMAO and vasculopathy in patients with kidney dysfunction has been previously studied. In patients with estimated glomerular filtration rates of less than 45 mL/min/1.73 m^2^, the highest tertile of serum TMAO was associated with more severe coronary vasculature lesions [38]. In patients with ESKD undergoing hemodialysis, serum TMAO concentration was independently positively associated with calcification of the abdominal aorta [39]. Among patients with CKD, targeted TMAO reduction through probiotic administration and direct inhibition of TMAO synthesis are potential therapeutic strategies for atherosclerotic disease and vascular calcification [40].

We did not measure TMAO levels in the peritoneal dialysate fluid. Since TMAO is a small, water-soluble molecule, it can readily pass through the dialyzer or peritoneal membrane, and a single hemodialysis session can effectively remove a substantial amount of TMAO [41]. However, no studies to date have examined the concentration of TMAO in peritoneal dialysate fluid. Therefore, whether TMAO levels in PD dialysate fluid are associated with AS or other CV outcomes remains unclear.

This study also showed that advanced age, diabetes, and high blood pressure were independently associated with high cfPWV in patients undergoing chronic PD. These variables are well-known traditional risk factors for AS and overt CV diseases [42,43,44]. Furthermore, waist circumference was an independent predictor of AS and could be used to differentiate AS from non-AS in PD patients. Prior studies on relatively healthy volunteers and participants with chronic medical illnesses consistently have shown that AS was correlated with waist circumference, visceral fat area, waist-to-hip ratio, and waist-to-height ratio [45,46,47]. Excess adiposity in visceral tissues leads to glucose intolerance, sustained inflammation, and vascular dysfunction [48,49]. Recognizing that various clinical and biological factors influence AS, we agree that achieving strong predictive power (AUC > 0.8) with a single biomarker may be challenging. To address this, we generated an AUC curve by incorporating clinical variables with significant differences from Table 1 (age, BUN, eGFR, and UPCR), which improved the AUC from 0.712 to 0.807. We included Figure 1 to illustrate this result. In the limitations section, we also pointed out that an AUC of 0.712 for myostatin represents only an acceptable predictive value, suggesting that additional biomarkers with higher predictive capacity, or a combination of biomarkers, would be necessary to enhance clinical utility for predictive purposes. Notably, biomarkers of mineral disorders (i.e., calcium, phosphorus, and iPTH levels) were not correlated with central AS in this study.

There were limitations in this research. First, the small sample size and the restriction of patient recruitment to certain centers limited the external validity of this clinical study. Second, the cross-sectional design made it difficult to infer a causal relationship between TMAO and AS. Third, we measured the serum TMAO level of each participant only once. Fourth, although inflammation may have been involved in the atherogenic process, we did not collect data on inflammatory markers, such as C-reactive protein and interleukins. Further longitudinal studies with larger sample sizes will help us clarify the relationship between C-reactive protein and interleukins and AS in kidney failure patients undergoing long-term PD. Fifth, in addition to the inflammatory biomarkers, the study did not consider other potential confounders, including the extent of hypervolemia and the load of dextrose and advanced glycation end-products.

## 4. Conclusions

High serum TMAO concentration was significantly associated with high cfPWV and independently predicted the presence of AS in patients with ESKD on long-term PD treatment. Future longitudinal trials on a larger number of patients will strengthen the results of this study and confirm the causative role of TMAO in vascular stiffness and CV consequences.

## 5. Materials and Methods

### 5.1. Study Participants

The study with a cross-sectional design was performed at four hospitals belonging to the Tzu Chi Medical Foundation in Taiwan. The study protocol was evaluated and approved by the research ethics committee of Hualien Tzu Chi Hospital, Buddhist Tzu Chi Medical Foundation (approval number: IRB108-219-A). From February 2020 to May 2021, we enrolled 160 patients on maintenance PD for ESKD. Only PD patients of the attending physicians of nephrologists participating in the study were included in the case enrollment. Each participant was provided with a written informed consent form. The exclusion criteria were an ongoing infectious disease, acute coronary syndromes, acute decompensated heart failure, cerebrovascular accident, active malignant tumor, prior limb amputation, and refusal to provide informed consent.

Data on age, sex, causes of ESKD, total duration of PD therapy, modality of PD, and prescription of chronic antihypertensive and lipid-lowering medications were reviewed from the medical records. Small solute clearance indicators were also retrieved from medical charts, including overall and peritoneal weekly creatinine clearance and weekly Kt/V. The most recent PD adequacy data for all patients were based on the latest data checked prior to the case filing date. The residual kidney function was assessed through the 24-h creatinine clearance of urine, in accordance with the recommendations from the Taiwan Society of Nephrology.

After asking each patient to rest in a sitting position for 10 min, blood pressure was measured by trained staff members using a regularly calibrated mercury sphygmomanometer and an appropriately sized cuff. A diagnosis of hypertension was based on an SBP of ≥140 mm Hg, a DBP of ≥90 mmHg, and/or intake of antihypertensives in the previous 2 weeks. Patients were diagnosed as having DM if their fasting plasma glucose exceeded or was equal to 126 mg/dL and/or they were chronically receiving antidiabetic therapy.

### 5.2. Anthropometric and Biochemical Investigations

BMI was calculated as the body weight (kg) divided by the height squared (m^2^). With the patients standing erect and at the end of exhalation, we measured the waist circumference three times at the midpoint between the top of the iliac crest and the lower margin of the rib cage; the average of the results was recorded.

A morning fasting blood sample (5 mL) was obtained from each participant after the complete drainage of the dialysate and before daytime PD fluid instillation. A small proportion of the blood sample was used for complete blood count (Sysmex SP-1000i; Sysmex American, Mundelein, IL, USA). The remaining 4.5 mL of blood specimen promptly underwent centrifugation at 3000× *g* for 10 min. Using an autoanalyzer, serum was collected for biochemical estimation of total cholesterol, glucose, albumin, blood urea nitrogen, creatinine, total calcium, and phosphorus concentrations (Siemens Advia 1800; Siemens Healthcare, Henkestr, Germany). Enzyme-linked immunosorbent assay kits (IBL International GmbH, Hamburg, Germany) measured serum iPTH.

### 5.3. High-Performance Liquid Chromatography-Mass Spectrometry for Determining Serum Trimethylamine N-Oxide Concentration

The high-performance liquid chromatography (HPLC) unit (Waters e2695 Separations Module, Waters Corporation, Milford, MA, USA), which contained a Phenomenex Luna^®^ C18(2) column (5 µm, 250 × 4.60 mm, 100 Å, Phenomenex, Torrance, CA, USA), was connected to a quadrupole mass spectrometer (ACQUITY QDa, Waters Corporation, Milford, MA, USA). Trimethylamine-d9 *N*-oxide (d9-TMAO) was used as the internal standard for estimating TMAO levels. For the HPLC component, the column temperature was controlled at 40 °C, and the flow rate was 0.8 mL/min for the mobile phase. The initial mobile-phase solution containing 95% eluent A (0.1% formic acid in water) and 5% eluent B (0.1% formic acid in methanol) was maintained for 1 min. In the subsequent 12 min, gradient elution was adopted to linearly increase the proportion of eluent B from 5% to 70%; 70% eluent B was maintained for two additional minutes. During column re-equilibration, the fraction of eluent B was decreased to 50%. During mass spectrometry, electrospray ionization produced ions in the gaseous phase. TMAO and d9-TMAO were continuously monitored in the positive-ion mode; the ratios of the mass number to the charge number were 76.0 *m*/*z* for TMAO and 85.1 *m*/*z* for d9-TMAO. Empower^®^ 3.0 software (New York, NY, USA) was used for data processing and integration [23,24].

### 5.4. Measurements of Carotid–Femoral Pulse Wave Velocity as a Marker of Central AS

The pulse wave velocity was checked with a SphygmoCor XCEL device (AtCor Medical, Sydney, NSW, Australia). The tonometer, femoral cuff, and tubing were authenticated using the SphygmoCor XCEL system to improve accuracy. The troubleshooting, calibration, and quality control measures complied with the illustrations from the manual [24]. Patients were ordered to rest in recumbency for approximately 10 min before the procedure was initiated and to relax and avoid talking or limb movements throughout the measurements. The SBP and DBP of the brachial arteries were measured by sphygmomanometry after cuff inflation, and the values were recorded. Subsequently, the cuff was reinflated within 5 s to capture the arterial waveforms. Trained staff palpated the patient’s carotid pulse and placed the tonometer tip on the pulsation point. After detecting carotid waveform regularity, the SphygmoCor XCEL system automatically inflated the femoral cuff around the thigh to simultaneously display femoral waveforms. We adopted the subtraction method to estimate the pulse traveling distance, which was defined as the difference between the length from the carotid pulse to the sternal notch and the length from the sternal notch to the femoral cuff. The cfPWV was eventually calculated as the pulse traveling distance divided by the carotid–femoral pulse transit time and expressed in m/s.

Each patient underwent two cfPWV measurements, and the average value was recorded. Based on the clinical guideline definition [50], patients with a cfPWV value of ≥10 m/s were assigned to the AS group.

### 5.5. Statistical Analysis

We performed the Kolmogorov–Smirnov test to check whether the continuous variables had normality. The variables showing normality were expressed as mean and standard deviation and compared between the AS and control group using the unpaired Student’s *t*-test. Parameters without normal distribution were compared using the Mann–Whitney U test. Qualitative variables were shown as numbers and percentages and were differentiated between groups using the chi-square test. Variables with significant differences (age, diabetes, waist circumference, SBP, fasting glucose, weekly Kt/V, and TMAO) were included as adjusted factors in the multivariate logistic regression analysis to identify the independent predictors of AS. Simple and multivariate regression analyses were used to determine the factors that were independently correlated with cfPWV; variables that showed skewness (i.e., PD treatment vintage, fasting glucose, iPTH, TMAO, and weekly creatinine clearance) were logarithmically transformed in advance. The statistical analyses were performed using IBM SPSS Statistics for Windows version 19.0 (IBM, Armonk, NY, USA). An ROC curve analysis (MedCalc Statistical Software version 23.0.9, MedCalc Software Ltd., Ostend, Belgium) was applied to determine the optimal cutoff of serum TMAO level for distinguishing AS from non-AS and for boxplot and scatter plot pictures.

## Figures and Tables

**Table 1:** Clinical features of those who underwent peritoneal dialysis in the arterial stiffness group (cfPWV > 10.0 m/s) or control group (cfPWV < 10.0 m/s).

Characteristic	All Participants (*n* = 160)	Control Group (*n* = 122)	Arterial Stiffness Group (*n* = 38)	*p* Value
Age (years)	56.51 ± 13.59	54.84 ± 13.97	61.87 ± 10.77	0.005 *
Peritoneal dialysis vintage (months)	46.34 (21.03–84.78)	43.30 (20.07–84.42)	51.96 (25.89–104.31)	0.337
Body mass index (kg/m^2^)	24.92 ± 4.15	24.68 ± 4.20	25.68 ± 3.96	0.194
Waist circumference (cm)	91.30 ± 11.09	89.95 ± 11.33	95.63 ± 9.11	0.005 *
Carotid–femoral PWV (m/s)	9.02 ± 1.61	8.34 ± 1.02	11.23 ± 1.09	<0.001 *
Systolic blood pressure (mmHg)	146.58 ± 20.97	144.48 ± 21.04	153.32 ± 19.54	0.023 *
Diastolic blood pressure (mmHg)	85.88 ± 14.08	85.82 ± 14.26	86.05 ± 13.66	0.929
Hemoglobin (g/dL)	9.62 ± 1.48	9.64 ± 1.42	9.57 ± 1.69	0.817
Total cholesterol (mg/dL)	165.89 ± 36.88	167.08 ± 37.55	162.11 ± 34.85	0.470
Fasting glucose (mg/dL)	101.00 (91.00–118.50)	99.50 (89.00–113.00)	108.00 (95.00–147.25)	0.006 *
Albumin (mg/dL)	3.60 ± 0.35	3.61 ± 0.34	3.57 ± 0.36	0.538
Blood urea nitrogen (mg/dL)	62.95 ± 20.38	62.26 ± 20.18	65.16 ± 21.12	0.446
Creatinine (mg/dL)	11.01 ± 3.04	11.02 ± 3.05	10.98 ± 3.04	0.952
Total calcium (mg/dL)	9.60 ± 0.74	9.57 ± 0.79	9.70 ± 0.55	0.350
Phosphorus (mg/dL)	5.27 ± 1.35	5.37 ± 1.40	4.94 ± 1.13	0.085
Intact parathyroid hormone (pg/mL)	257.11 (89.43–529.03)	257.11 (106.45–523.99)	241.65 (81.51–530.33)	0.754
TMAO (μg/L)	111.74 (72.04–165.20)	102.73 (68.87–144.91)	168.73 (121.43–246.18)	<0.001 *
Weekly Kt/V	2.09 ± 0.44	2.13 ± 0.46	1.96 ± 0.37	0.045 *
Peritoneal Kt/V	1.86 ± 0.48	1.89 ± 0.50	1.77 ± 0.40	0.175
Total Clcr (L/week)	56.45 (48.13–69.45)	56.35 (47.70–69.58)	56.60 (50.95–67.08)	0.645
Peritoneal Clcr (L/week)	47.58 ± 13.42	46.95 ± 13.67	49.61 ± 12.54	0.288
Urine Clcr (L/week)	2.75 (0.00–19.82)	3.02 (0.00–20.86)	1.33 (0.00–18.39)	0.659
Female, *n* (%)	88 (55.0)	70 (57.4)	18 (47.4)	0.279
Diabetes, *n* (%)	62 (38.8)	39 (32.0)	23 (60.5)	0.002 *
Hypertension, *n* (%)	119 (74.4)	88 (72.1)	31 (81.6)	0.244
CAPD, *n* (%)	61 (38.1)	47 (38.5)	14 (36.8)	0.852
ARB use, *n* (%)	109 (68.1)	81 (66.4)	28 (73.7)	0.400
β-blocker use, *n* (%)	77 (48.1)	58 (47.5)	19 (50.0)	0.791
CCB use, *n* (%)	99 (61.9)	75 (61.5)	24 (63.2)	0.852
Statin use, *n* (%)	48 (30.0)	38 (31.1)	10 (26.3)	0.570
Fibrate use, *n* (%)	18 (18.0)	12 (17.1)	6 (20.0)	0.733
Etiology of ESKD				
Diabetes mellitus, *n* (%)	61 (38.1)	39 (32.0)	22 (57.9)	
Chronic glomerulonephritis, *n* (%)	54 (33.8)	43 (35.2)	11 (28.9)	
Hypertensive nephrosclerosis, *n* (%)	22 (13.7)	19 (15.6)	3 (7.9)	
Others, *n* (%)	23 (14.4)	21 (17.2)	2 (5.3)	

The continuous variables are expressed as mean ± standard deviation and median (interquartile range) for normal and non-normal distributions, respectively, and were separately analyzed using Student’s *t*-test and the Mann–Whitney U test. The categorical values are expressed as numbers (%) and further compared between the groups with the chi-square test. Abbreviations: CAPD, continuous ambulatory peritoneal dialysis; TMAO, trimethylamine *N*-oxide; weekly Kt/V, weekly fractional clearance index for urea; Clcr, clearance of creatinine; ARB, angiotensin-receptor blocker; CCB, calcium-channel blocker; ESKD, end-stage kidney disease. * *p* < 0.05 denotes statistical significance.

**Table 2:** Multivariable logistic regression analysis of the factors correlated to arterial stiffness.

Variables	Odds Ratio	95% Confidence Interval	*p*-Value
TMAO, 1 μg/L	1.011	1.005–1.017	<0.001 *
Age, 1 year	1.053	1.013–1.095	0.009 *
Waist circumference, 1 cm	1.047	1.003–1.094	0.038 *
Systolic blood pressure, 1 mmHg	1.020	0.997–1.044	0.084
Diabetes, present	1.233	0.415–3.660	0.707
Glucose, 1 mg/dL	0.996	0.981–1.012	0.651
Weekly Kt/V	0.449	0.152–1.321	0.146

The multivariable logistic regression analysis was performed by using the adopted factors which showed a significant difference between the arterial stiffness and non-arterial stiffness groups, namely age, diabetes, waist circumference, systolic blood pressure, fasting glucose, Weekly Kt/V, and TMAO. Abbreviations: TMAO, trimethylamine *N*-oxide; weekly Kt/V, weekly fractional clearance index for urea. * *p* < 0.05 was considered statistically significant.

**Table 3:** Correlation between carotid–femoral pulse wave velocity levels and clinical variables.

Variables	Carotid–Femoral Pulse Wave Velocity (m/s)				
	Simple Linear Regression		Multivariate Linear Regression		
	*r*	*p*-Value	Beta	Adjusted R^2^ Change	*p*-Value
Female	−0.144	0.070	-	-	-
Diabetes	0.368	<0.001 *	0.163	0.130	0.022 *
Hypertension	0.037	0.358	-	-	-
Age (years)	0.347	<0.001 *	0.218	0.071	<0.001 *
Log-PD vintage (months)	0.065	0.411	-	-	-
Body mass index (kg/m^2^)	0.240	0.002 *	-	-	-
Waist circumference (cm)	0.330	<0.001 *	0.256	0.058	<0.001 *
Systolic blood pressure (mmHg)	0.295	<0.001 *	0.208	0.047	0.002 *
Diastolic blood pressure (mmHg)	0.062	0.434	-	-	-
Hemoglobin (g/dL)	0.069	0.386			
Total cholesterol (mg/dl)	−0.130	0.101	-	-	-
Log-Glucose (mg/dL)	0.299	<0.001 *	-	-	-
Albumin (mg/dL)	−0.042	0.595	-	-	-
Blood urea nitrogen (mg/dL)	0.011	0.888	-	-	-
Creatinine (mg/dL)	0.005	0.954	-	-	-
Total calcium (mg/dL)	0.147	0.063	-	-	-
Phosphorus (mg/dL)	−0.124	0.120	-	-	-
Log-iPTH (pg/mL)	−0.045	0.570	-	-	-
Log-TMAO (μg/L)	0.346	<0.001 *	0.213	0.038	0.002 *
Weekly Kt/V	−0.157	0.648	-	-	-
Peritoneal Kt/V	−0.054	0.494	-	-	-
Log-Total Clcr (L/week)	0.076	0.339	-	-	-
Peritoneal Clcr (L/week)	0.115	0.147	-	-	-
Urine Clcr (L/week)	−0.044	0.580	-	-	-

Variables including the PD vintage, glucose, iPTH, total clearance of creatinine, and TMAO levels showed skewed distribution and therefore were log-transformed before the simple and multivariate linear regression analysis. The adopted factors in the multivariate stepwise linear regression analysis included diabetes, age, body mass index, waist circumference, systolic blood pressure, log-glucose, and log-TMAO. Abbreviations: PD, peritoneal dialysis; iPTH, intact parathyroid hormone; TMAO, trimethylamine *N*-oxide; weekly Kt/V, weekly fractional clearance index for urea; Clcr, clearance of creatinine. * *p* < 0.05 was considered statistically significant.

## Data Availability

The original contributions presented in this study are included in this article and Supplementary Material. Further inquiries can be directed to the corresponding authors.

## Supplementary Materials

The following supporting information can be downloaded at: https://www.mdpi.com/article/10.3390/toxins16120523/s1, Figure S1: The area under the receiver operating characteristic curve analysis for the waist circumference (A) and combined trimethylamine *N*-oxide and waist circumference (B) in distinguishing arterial stiffness and non-arterial stiffness in patients undergoing peritoneal dialysis. (C) The addition of waist circumference to trimethylamine *N*-oxide did not enhance the distinguishing power of the status of arterial stiffness.; Figure S2: The boxplots and scatter plots with regression lines between the variables (A) diabetes mellitus, (B) age, (C) waist circumference, (D) systolic blood pressure, and (E) log-TMAO and the carotid–femoral pulse wave velocity.
