# Supplementary material for: The Association Between Serum Trimethylamine N-Oxide and Arterial Stiffness in Chronic Peritoneal Dialysis Patients: A Cross-Sectional Study
Source: Toxins (Basel). 2024 Dec 3;16(12):523. doi: 10.3390/toxins16120523 (PMC11728722; doi:10.3390/toxins16120523)
Supplement: Supplementary file 1 [file toxins-16-00523-s001.zip › toxins-3266975-supplementary.pdf]

toxins

MDPI

Supplementary Materials

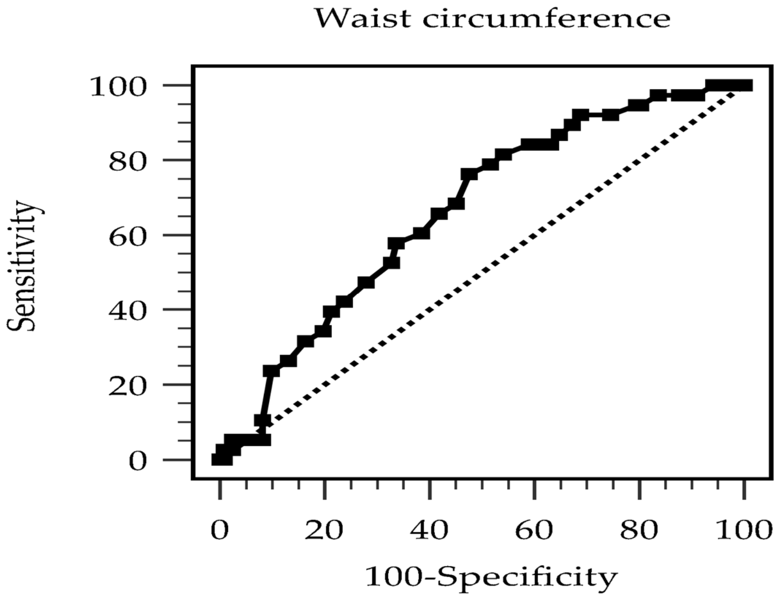

(A) The area under the curve was 0.665 (95% CI 0.586–0.737, $p = 0.0005$)

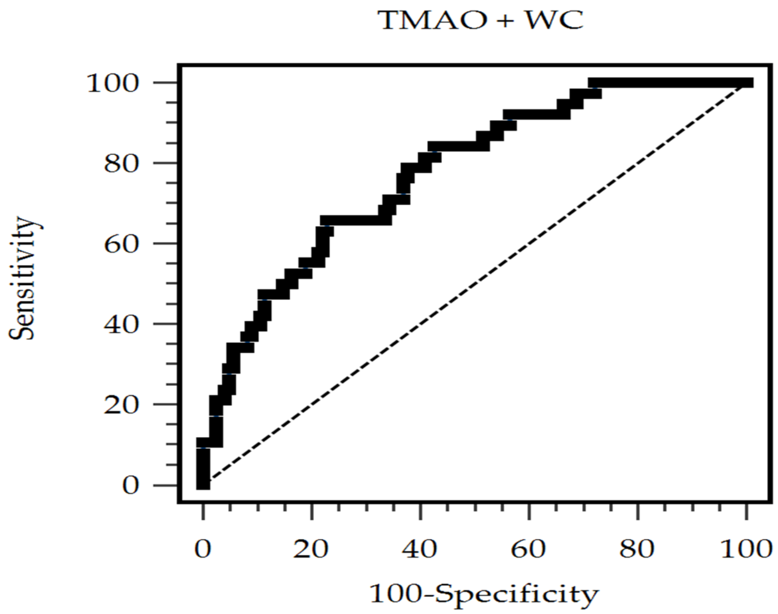

(B) The area under the curve was 0.774 (95% CI 0.702–0.837, $p < 0.0001$)

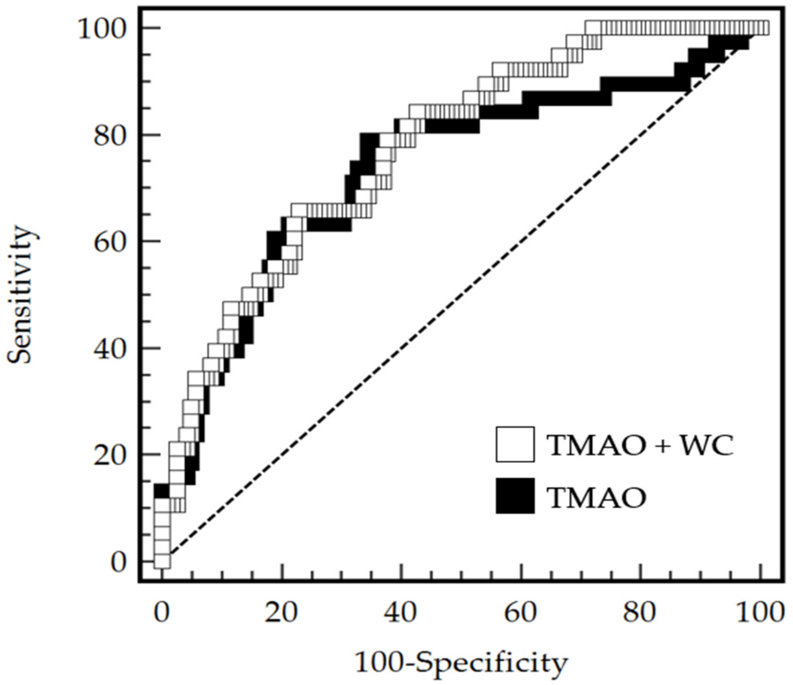

(C) The difference in the area under the curve between the trimethylamine *N*-oxide + waist circumference group to the trimethylamine *N*-oxide group was 0.0372 (95% CI −0.0124–0.0868, $p = 0.1416$).

Figure S1. The area under the receiver operating characteristic curve analysis for the waist circumference (**A**) and combined trimethylamine *N*-oxide and waist circumference (**B**) in distinguishing arterial stiffness and non-arterial stiffness in patients undergoing peritoneal dialysis. (**C**) The addition of waist circumference to trimethylamine *N*-oxide did not enhance the distinguishing power of the status of arterial stiffness. Abbreviations: TMAO, trimethylamine *N*-oxide; WC, waist circumference.

(A)

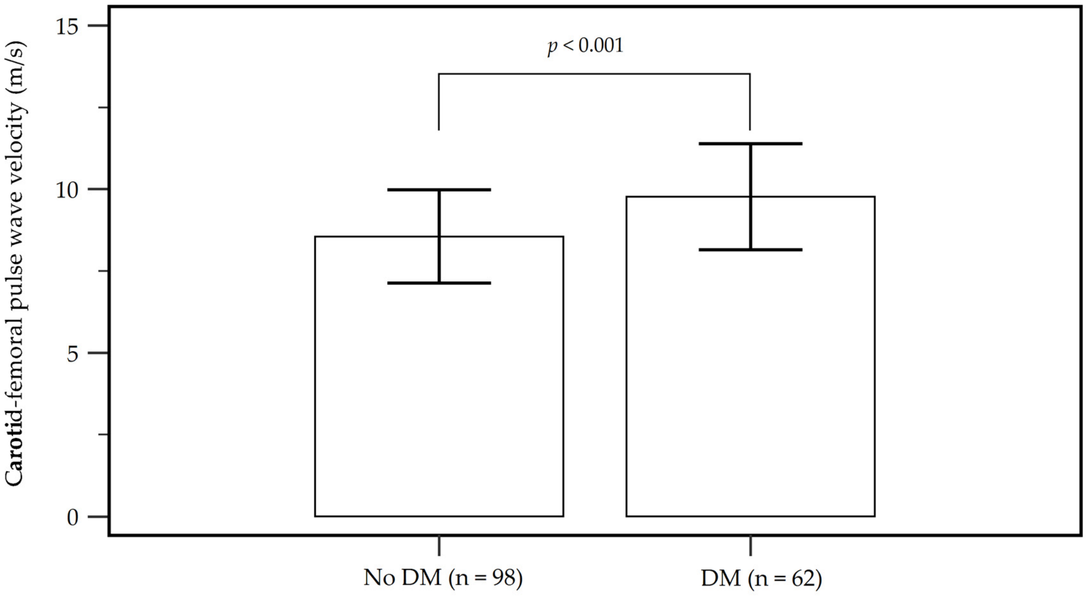

(B)

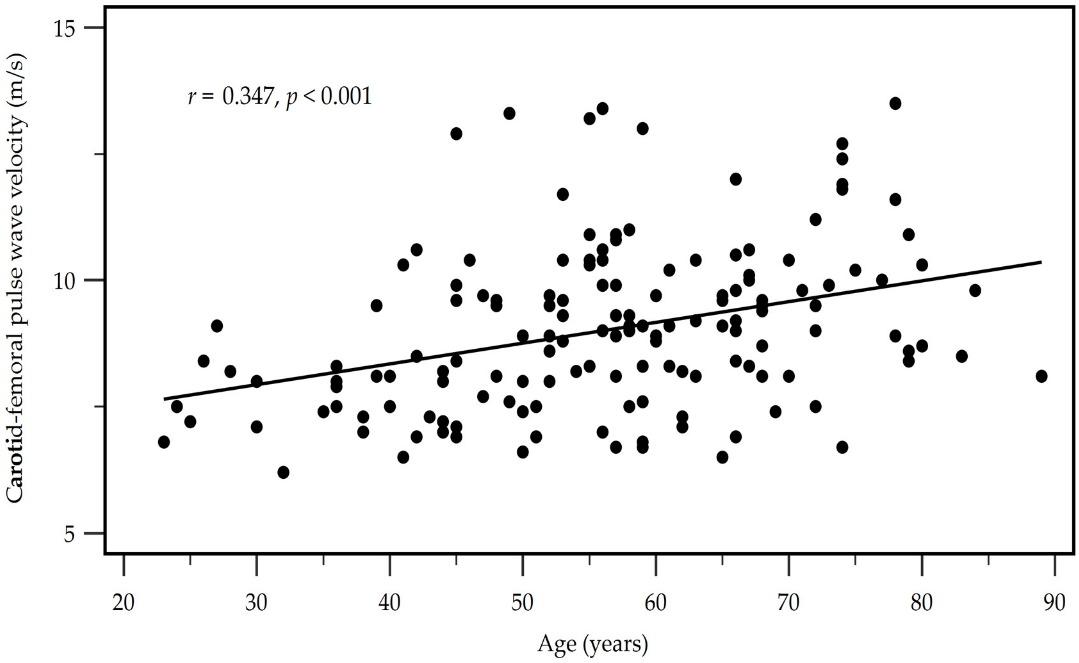

r = 0.347, p < 0.001
Carotid-femoral pulse wave velocity (m/s)
15
10
5
20
30
40
50
60
70
80
90
Age (years)

(C)

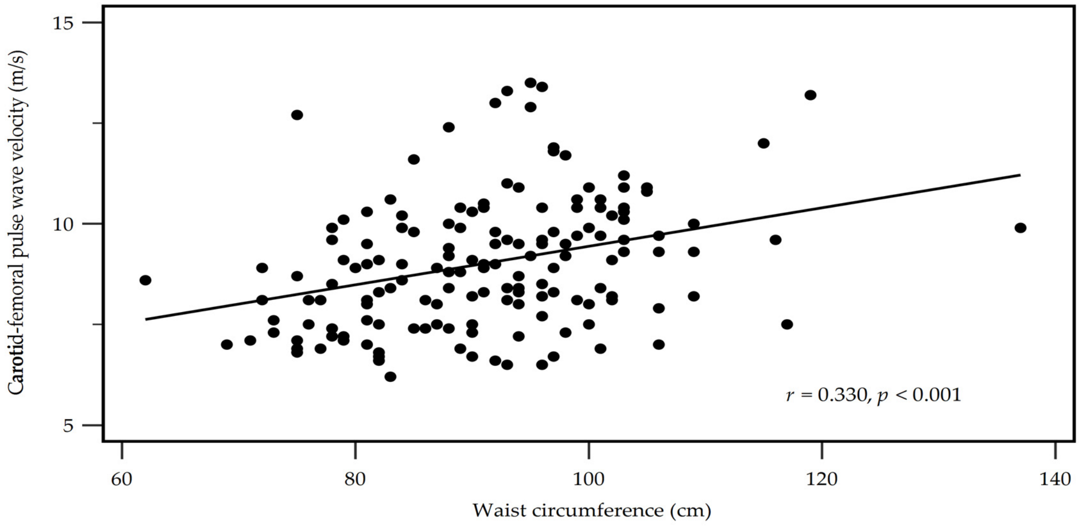

r = 0.330, p < 0.001
Carotid-femoral pulse wave velocity (m/s)
15
10
5
60
80
100
120
140
Waist circumference (cm)

(D)

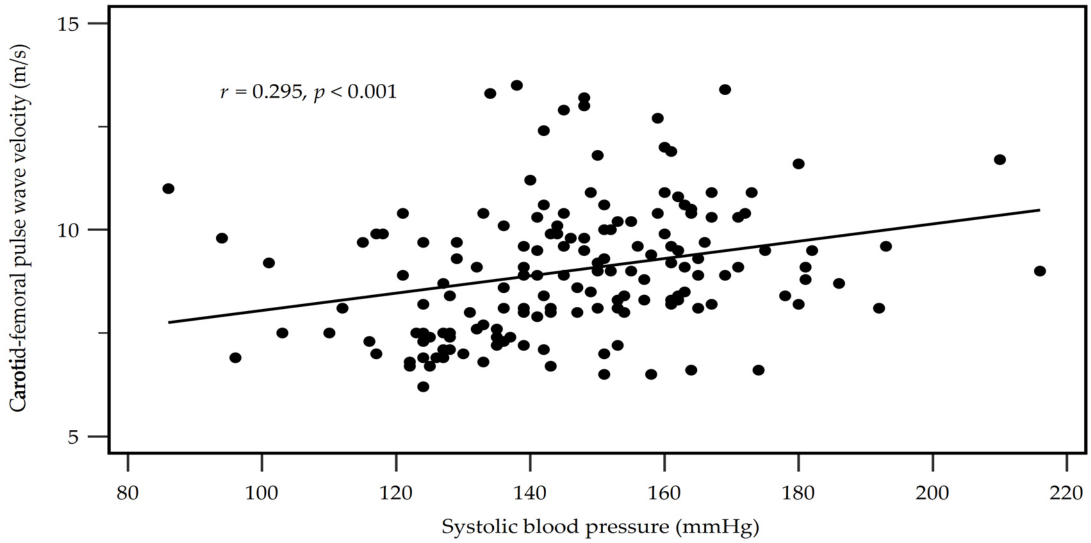

r = 0.295, p < 0.001
Carotid-femoral pulse wave velocity (m/s)
15
10
5
80
100
120
140
160
180
200
220
Systolic blood pressure (mmHg)

(E)

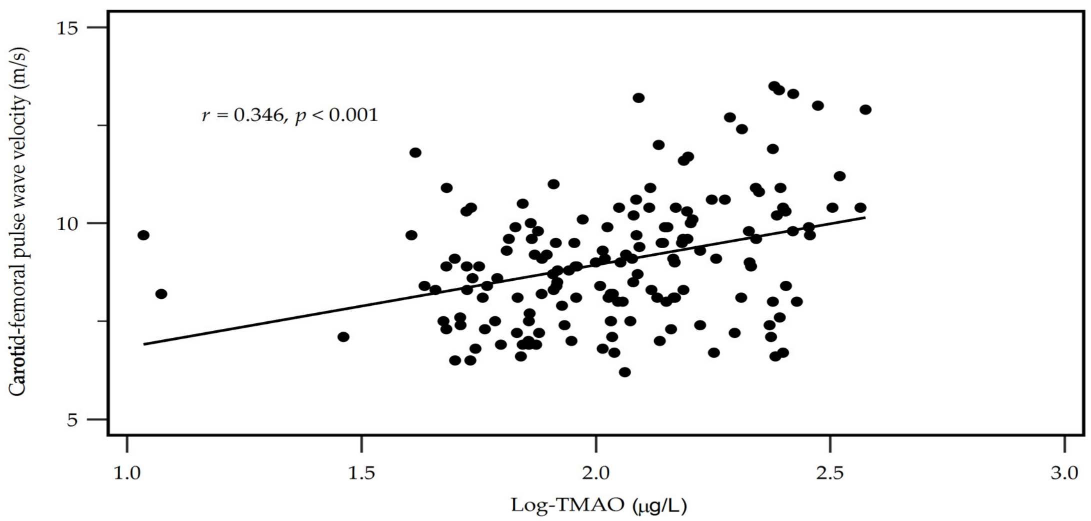

Figure S2. The boxplots and scatter plots with regression lines between the variables (**A**) diabetes mellitus, (**B**) age, (**C**) waist circumference, (**D**) systolic blood pressure, and (**E**) log-TMAO and the carotid-femoral pulse wave velocity. Abbreviations: DM, diabetes mellitus; TMAO; trimethylamine *N*-oxide.
